# Monocytes/macrophages activation contributes to b-gamma-glutamyltransferase accumulation inside atherosclerotic plaques

**DOI:** 10.1186/s12967-015-0687-6

**Published:** 2015-10-13

**Authors:** Eugenia Belcastro, Maria Franzini, Silvana Cianchetti, Evelina Lorenzini, Silvia Masotti, Vanna Fierabracci, Angela Pucci, Alfonso Pompella, Alessandro Corti

**Affiliations:** Department of Translational Research and New Technologies in Medicine and Surgery, Medical School, University of Pisa, Via Roma 55, 56126 Pisa, Italy; CITHEFOR-EA 3452, Faculté de Pharmacie, Université de Lorraine, Nancy, France; Department of Surgery and Medical, Molecular, and Critical Area Pathology, Medical School, University of Pisa, Pisa, Italy; Life Science Institute, Scuola Superiore Sant’Anna, Pisa, Italy; Histopathology Department, University Hospital, Pisa, Italy

**Keywords:** Gamma-glutamyltransferase, b-GGT fraction, Monocytes, Macrophages, Atherosclerosis

## Abstract

**Background:**

Gamma-glutamyltransferase (GGT) is a well-established independent risk factor for cardiovascular mortality related to atherosclerotic disease. Four GGT fractions have been identified in plasma, but only b-GGT fraction accumulates in atherosclerotic plaques, and correlates with other histological markers of vulnerability. The present study was aimed to evaluate whether macrophagic lineage cells may provide a source of b-GGT within the atherosclerotic plaque.

**Methods:**

GGT expression and release were studied in human monocytes isolated from peripheral blood of healthy donors. The growth factors GM-CSF and M-CSF were used to induce differentiation into M1-like and M2-like macrophages, respectively. Plaque GGT was investigated in tissue samples obtained from patients undergoing carotid endoarterectomy.

**Results:**

We found that M1-like macrophages express higher levels of GGT as compared to M2-like, and that both monocytes and M1-like macrophages—but not M2-like—are able to release the b-GGT fraction upon activation with pro-inflammatory stimuli. Western blot analysis of b-GGT extracted from plaques confirmed the presence of a GGT immunoreactive peptide coincident with that of macrophages.

**Conclusions:**

Our data indicate that macrophages characterized by a pro-inflammatory phenotype may contribute to intra-plaque accumulation of b-GGT, which in turn may play a role in the progression of atherosclerosis by modulating inflammatory processes and favouring plaque instability.

## Background

Monocyte/macrophage lineage cells represent one of the main cellular components of atherosclerotic lesions. Atherosclerosis is a progressive and chronic inflammatory disease characterized by cellular and molecular alterations within the arterial wall which lead to lumen narrowing and/or to plaque rupture with superimposed acute thrombosis [1, 2]. Such condition is by far the most frequent underlying cause of coronary, carotid and peripheral arterial disease [3]. The inflammatory reactions taking place in plaques implicate highly complex processes that are still not completely understood. However, it is known that the recruitment of leukocytes play a key role in these events, and monocytes in particular invade atherosclerotic lesions and differentiate into macrophages. The heterogeneity of macrophages detectable in atherosclerotic lesions has been a topic of great interest lately, with particular reference to the two major macrophage subpopulations involved in pro-inflammatory processes (“M1” phenotype) and in resolution and repair (“M2” phenotype), respectively. Actually, M1 and M2 macrophages are thought to represent the extreme polarization phenotypes of a continuum of pro- and anti-inflammatory macrophages all simultaneously present in atherosclerotic lesions [4–6]. M1 macrophages were demonstrated to be the predominant phenotype in rupture prone shoulder regions of human plaques, while M2 macrophages-associated markers were predominant in the adventitia [7]. On the other hand M2 macrophages were identified in more stable cell-rich areas, away from the lipid core of human atherosclerotic plaques [8, 9]. It was suggested that—in response to the changing environment in developing atherosclerotic plaques—macrophages may shift transiently between different phenotypes. Nevertheless a number of questions remain to be answered, e.g. little is known about the propensity of individual macrophage phenotypes to become foam cells, even if some reports suggest that both M1 and/or M2 macrophages may be foam cell precursors [5, 10].

Epidemiological studies showed that total serum gamma-glutamyltransferase (GGT) activity is an independent risk factor for cardiovascular mortality related to atherosclerotic disease [11] and a correlation between plasma GGT levels and the presence of multivessel disease has been proposed [12]. We recently identified four different GGT fractions in human blood [13] and an association between the fraction with highest molecular weight (b-GGT) with known cardiovascular risk factors—such as LDL-cholesterol, triglycerides, glucose and C-reactive protein—was demonstrated [14]. More recently, a first characterization revealed that b-GGT consists of membrane microvesicles with physical properties resembling those of exosomes [15].

Importantly, b-GGT has been detected also inside atherosclerotic plaques [16] and it has been suggested that this may be a factor in pathogenesis of atherosclerosis. Indeed, GGT enzyme activity is involved in the modulation of inflammatory mediators such as leukotrienes [17] and S-nitrosoglutathione (GSNO) [18] and it has been repeatedly documented that in the presence of glutathione and transition metal cations (in particular Fe^3+^ and Cu^2+^), GGT activity is able to trigger the production of reactive oxygen species (superoxide, hydrogen peroxide), thus promoting LDL oxidation and other pro-oxidant reactions potentially involved in the progression of atherosclerotic lesions [19–21]. The necrotic core of the plaque, where both GGT [19, 20] and iron [22] are present at adequate concentrations, represents an environment favourable to the evolution of such reactions.

Human mononuclear cells have long been known to express GGT activity [23, 24] and GGT-positive CD68+ macrophage-derived foam cells are present in the intimal layers of human atherosclerotic plaques [25, 26]. The levels of intra-plaque b-GGT activity were demonstrated to correlate with both plasma b-GGT and levels of macrophagic infiltration, a histological marker of plaque vulnerability [27]. Against this background, the aim of the present study was to assess whether monocytes/macrophages may represent a source of b-GGT within the atherosclerotic plaque.

## Methods

### Chemicals

Unless otherwise indicated, all reagents were from Sigma Chemical Co. (St. Louis, MO, USA). Recombinant human cytokines and growth factors were purchased from PeproTech (London, UK).

### Ethics, consent and permissions

The study was approved by the Institutional Ethics Committee of the University Hospital of Pisa, and conformed to the Declaration of Helsinki. Patients gave their verbal informed consent to participate and the data were analysed anonymously.

### Isolation and activation of monocytes

Human peripheral blood mononuclear cells (PBMCs) were isolated by Histopaque^®^-1077 density centrifugation from the blood of healthy donors. Monocytes were enriched by plating (10 × 10^6^ cells/wells) PBMCs in 6-well plates (Sarstedt) with RPMI 1640 medium containing 2 mmol/L l-glutamine and 10 % v/v fetal calf serum, the latter ultracentrifuged (100,000×*g*, 120 min, 4 °C) before addition to remove serum-associated b-GGT (data not shown). After 2 h, non-adherent cells were removed and adherent cells were washed five times with RPMI 1640. Cytospin preparations of trypsinized cells were prepared to evaluate the purity of adherent monocytes (74.5 % ± 3.4 monocytes, 25.0 % ± 3.6 lymphocytes, 1.1 % ± 0.4 neutrophils; n = 8). Monocytes-enriched cell preparations were then incubated with ionomycin (1 μM, 15 min), LPS (5 μg/ml, 24 h) or a combination of TNFα/IL-1β (both 10 ng/ml, 24 h), as described by others [28–30]. All cells were kept in a humidified incubator with 5 % CO_2_/95 % air and cell viability was assessed by Trypan blue exclusion. Finally media were collected and centrifuged at 300×*g* (5 min, 4 °C) and 10,000×*g* (10 min, 4 °C) before GGT determinations. Further details are reported in the figure legends.

### Polarization of monocytes into M1-like or M2-like phenotype

*In vitro* differentiation of monocytes into macrophages with properties similar to M1 and M2 cells (“M1-like” and “M2-like”, respectively) was obtained as repeatedly described [10, 31–34]. Briefly, PBMCs isolated by Histopaque^®^-1077 density centrifugation were left to adhere for 24 h in RPMI 1640 medium integrated with 2 mmol/L l-glutamine, 100 U/ml penicillin, 100 μg/ml streptomycin and 10 % v/v fetal calf serum. After 24 h, non-adherent cells were removed, while adherent cells were cultured for additional 6 days in complete medium integrated with 50 ng/ml recombinant human granulocyte/macrophage colony stimulating factor (GM-CSF) or with 50 ng/ml macrophage colony stimulating factor (M-CSF), respectively. Where indicated, cells were incubated for additional 24 h with a combination of TNFα/IL-1β (both 10 ng/ml) [28–30] and media were collected as described above.

In another set of experiments, adherent monocytes were incubated with M-CSF for 6 days in the presence of a mouse anti-IL10 antibody (1 μg/ml; Abcam) or the corresponding isotype control (1 μg/ml; Abcam). Other experiments were finally performed by incubating adherent monocytes with TNFα (10 ng/ml) and/or IL-10 (20 ng/ml) in complete medium for 48 h. Where indicated, cells were pre-treated (20 min) with IL-10, then TNFα was added to incubation media. Cell viability was assessed by Trypan blue exclusion. Further details are reported in the figure legends.

### Plaques and monocytes staining

Samples of carotid plaques were from patients undergoing carotid endarterectomy at the General and Vascular Surgery (University Hospital, Pisa, Italy) and were already fully characterized [27]. The patients were asymptomatic, since endarterectomy is recommended in case of severe carotid artery stenosis (>70 %) even in absence of neurological symptoms [35]. Surgically excised carotid plaques were collected on ice and dissected into 5-mm segments, then a segment of the plaque was frozen at −20 °C in Optimal Cutting Temperature (OCT) medium for in situ evaluation of enzymatic activity. Plaque sections were stained as described [27]. Briefly, serial 3 μm paraffin sections were stained with hematoxylin-eosin and Masson’s trichrome method. Immunohistochemistry was performed on adjacent paraffin-embedded sections. Macrophages were identified by immunoperoxidase staining by using a specific monoclonal antibody raised against CD68 antigen (Dakopatts, Glostrup, Denmark) and the immunoreaction was visualized by 3-diaminobenzidine substrate. GGT immunostaining was performed by using a previously characterized polyclonal antibody directed against the heavy chain of human GGT antibody at the appropriate dilution (1:1600) [36]. Basing on plaque histology [27], two (#1, #2) representative thin-cap fibroatheromas with large necrotic core and medium–high macrophage infiltration score, and one (#3) stable, thick-cap fibroatheroma with small necrotic scores and low macrophage infiltration score, were selected (Table 1).

**Table 1:** Histological features of the selected plaques used

N.	Cap thickness	Necrotic core	CD68+ macrophages	Fibrous tissue	Calcification	Intra-plaque activities (mU/g tissue)	
						b-GGT	Total GGT
#1	Thin and ulcerated	High	Medium	Low	Low	28.89	100.73
#2	Thin and ulcerated	High	Medium	Low	Low	14.61	47.02
#3	Thick	Medium	Low	Medium	High	9.43	35.58

Cytochemical staining for GGT activity was performed on air-dried buffy coat films fixed in a phosphate-buffered acetone formaldehyde mixture (PBAF) and incubated with GGT substrate gamma-glutamyl-4-methoxy-2-naphtylamide and Fast Garnet GBC, as previously described [24]. Nuclei were counterstained with methyl green.

### Microvesicles isolation

Portions of plaque tissue were snap-frozen in liquid nitrogen, mechanically disrupted and homogenized in 5 % volume of cold hypotonic buffer (PBS diluted 10 folds) using a Potter homogenizer. Isolation of microparticles and exosomes from monocytes/macrophages supernatants as well as from homogenates of atherosclerotic lesions was performed by a differential centrifugation procedure [15]. Samples were first centrifuged at 2000×*g* for 10 min at 4 °C, then supernatants were centrifuged at 10,000×*g* (45 min, 4 °C) to remove large debris. Again, supernatants were collected, transferred into ultracentrifuge tubes and centrifuged at 100,000×*g* (120 min, 4 °C). Pellets were then washed (100,000×*g*, 120 min, 4 °C) and finally resuspended in physiological saline.

### Western blot analysis

For western blot determinations of GGT, isolated monocytes, lymphocytes, platelets and cultured endothelial HMEC-1 cells—harvested in hypotonic lysis buffer (10 mM Tris–HCl, pH 7.8)—or plaque derived exosomes were used. Non-adherent lymphocytes were isolated from plated PBMCs, whereas platelets were obtained from buffy-coat derived platelet concentrates. All samples were thoroughly washed in order to remove contaminating plasma GGT. Endothelial HMEC-1 cells were grown in MCDB 131 medium (Life Technologies) supplemented with 1 µg/ml hydrocortisone, 10 ng/ml EGF (Life Technologies) and 10 % v/v fetal calf serum, and cultured at 37 °C in a 5 %/95 % CO_2_/air atmosphere.

All samples were separated by 8 % SDS-PAGE [37] and incubated with rabbit anti-GGT IgG directed against the C-terminal 20 amino acids of human GGT heavy chain prepared as described [36]. Visualization of protein bands was obtained using a horseradish peroxidase-conjugated anti-rabbit IgG antibody (Santa Cruz Biotechnology, Santa Cruz, CA, USA) and the ECL detection system (Roche, Basel, Switzerland). Bands were analyzed with a Bio-Rad ChemiDoc apparatus equipped with the QuantityOne software.

### Fractional GGT analysis by high-performance gel filtration chromatography

Determination of GGT fractions was performed as previously described [13, 38] by a FPLC system (AKTA-purified-10, GE-Healthcare). Separation and quantification of GGT fractions was performed by gel-filtration chromatography (Superose 6 10/300, GE Healthcare) followed by post-column injection of the fluorescent substrate gamma-glutamyl-7-amido-4-methylcoumarin. Intensity of the fluorescence signal was expressed in arbitrary fluorescence units (f.u.) and the area under chromatographic peaks was proportional to GGT activity. The elution volume for b-GGT fraction is 12.9 ml, corresponding to a molecular weight (MW) of 2000 kDa [13].

### Other determinations

GGT activity was determined according to Huseby and Strömme [39]. Cytokines were measured using specific enzyme-linked immunosorbent assay kits (TNF-α, R&D Systems; IL-6 and IL-10, Affymetrix eBioscience) according to the manufacturer’s instructions. Protein content was determined by the Bradford’s method using the Bio-Rad protein assay reagent. Statistical analysis of data was performed by Student’s *t* test for paired observations and one-way ANOVA with Newman–Keuls test for multiple comparisons.

## Results

### Monocytes activation

**Fig. 1:**
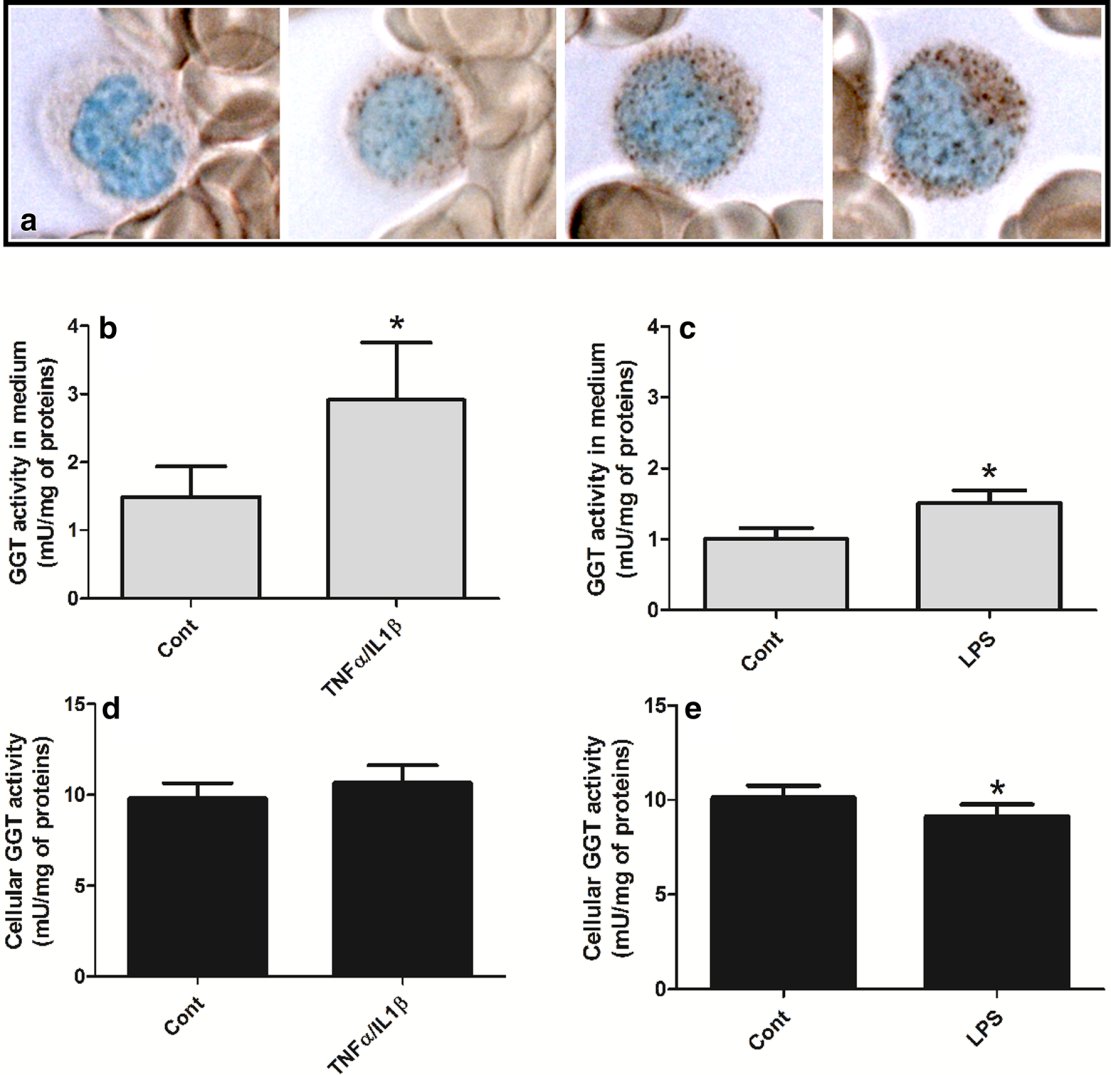
Cytochemical staining for GGT enzyme activity expressed in activated monocytes. Monocytes from the blood of healthy donors expressing different levels of GGT activity. The azo-dye reaction product (*reddish-brown*) varies in intensity from slight to very strong, resulting in diffuse staining or granular positivity. Nuclei were counterstained with methyl green. Original magnification: ×100 (**a**). Monocytes isolated from fresh buffy coats were incubated in the presence of TNFα/IL-1β (both 10 ng/ml; 24 h) or LPS (5 μg/ml; 24 h). GGT activity was measured in the 10,000×*g* centrifuged supernatants (**b**, **c**) and in cellular homogenates (**d**, **e**). Results—expressed as mU/mg of cellular proteins—are mean ± SD of five to nine separate determinations. Data were analyzed by Student’s t test for paired data; *p < 0.05 as compared with the corresponding control

In agreement with earlier evidence [24], different levels of GGT activity were detected when monocytes isolated from the blood of healthy donors were stained for the enzyme (Fig. 1a), and a mean value of 10.1 ± 2.0 mU/mg of protein in the whole homogenate was calculated. In order to understand whether such cells were able to release GGT, isolated monocytes were exposed to activating substances and GGT activity was measured in the incubation media. As reported in Fig. 1, both the combination of pro-inflammatory cytokines TNFα/IL-1β (Fig. 1b) and bacterial lipopolysaccharide (LPS; Fig. 1c) significantly increased (p < 0.05) GGT activity in the incubation media as compared to controls. In a separate set of experiments also the calcium ionophore ionomycin induced a significant (p < 0.05) release of GGT activity in the incubation media (data not shown). GGT activity was also detected in the media of the corresponding control samples (Fig. 1b, c), possibly ensuing from a weak activation of monocytes during isolation/incubation procedures. As regard cell-associated GGT activity, TNFα/IL-1β treatment did not produce any appreciable effect (Fig. 1d), whereas a significant decrease was induced by LPS (Fig. 1e; p < 0.05).

### Macrophages activation

**Fig. 2:**
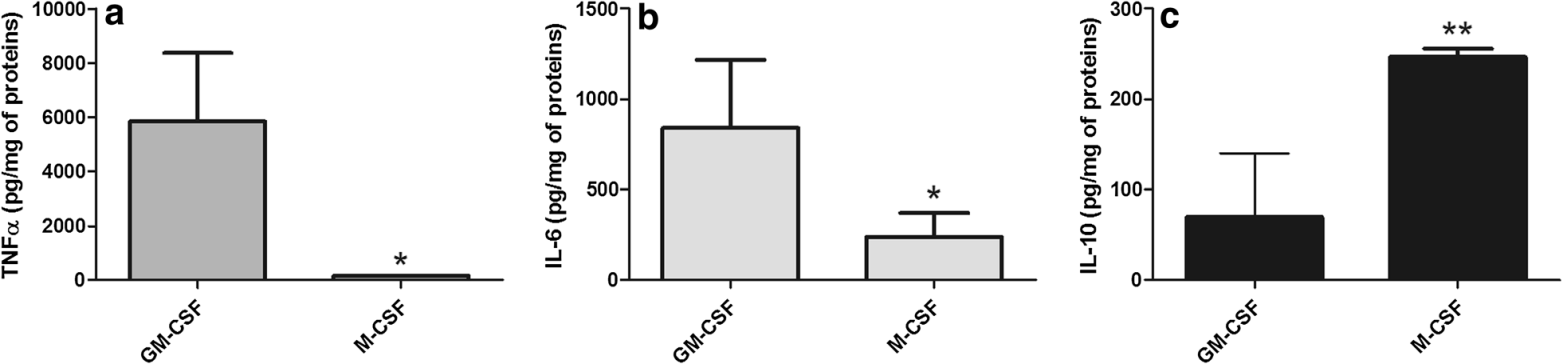
Cytokines in the supernatant of monocyte-derived macrophages. Monocytes isolated from fresh buffy coats were incubated for 6 days in complete medium in the presence of GM-CSF (50 ng/ml) or M-CSF (50 ng/ml) in order to induce the differentiation into M1-like or M2-like macrophages, respectively. Data represent levels of **a** TNFα, **b** IL-6 and **c** IL-10 in culture media on the sixth day of incubation. Results are mean ± SD of five separate determinations. Data are expressed as mU/mg of cellular proteins and were analyzed by Student’s t test for paired data; *p < 0.01 and **p < 0.05 as compared with the corresponding control

**Fig. 3:**
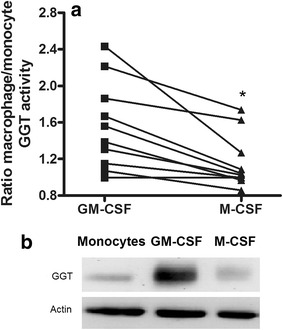
Effects of GM-CSF and M-CSF exposure on cellular GGT expression. Monocytes isolated from fresh buffy coats were incubated for 6 days in complete medium in the presence of GM-CSF (50 ng/ml) or M-CSF (50 ng/ml) in order to induce the differentiation into M1-like or M2-like macrophages, respectively. **a** Data are *ratioes* between GGT activities determined after differentiation and activities present in initial monocytes. The results of ten separate paired incubations are shown. *Solid lines* connect data pairs obtained with the same starting monocytes preparation. Statistical analysis was performed by Student’s t test for paired data; *p = 0.0016. **b** Representative western blot analysis of GGT expression. *Lane 1* monocytes; *lane 2* M1-like macrophages; *lane 3* M2-like macrophages

M1- and M2-like macrophages were obtained by incubating aliquots of monocytes (isolated from the same buffy coat) with GM-CSF or M-CSF, respectively. As previously described [32], after 6 days of culture the majority of M1-like macrophages presented with a classical adherent, “fried egg” morphology, whereas M2-like macrophages primarily presented with a stretched, spindle-like morphology (data not shown). The analysis of cytokines concentration in culture media revealed significantly (p < 0.01) higher levels of TNFα and IL-6 (Fig. 2a, b) and lower (p < 0.05) levels of IL-10 (Fig. 2c) for M1-like as compared to M2-like macrophages. M1-like macrophages showed also significantly (p < 0.005) higher levels of cell associated GGT activity (Fig. 3a) and protein (Fig. 3b) as compared to M2-like macrophages. SDS-PAGE analysis revealed that the GGT heavy subunit presented with the same apparent MW in both differentiated macrophages and monocytes (Fig. 3b).

**Fig. 4:**
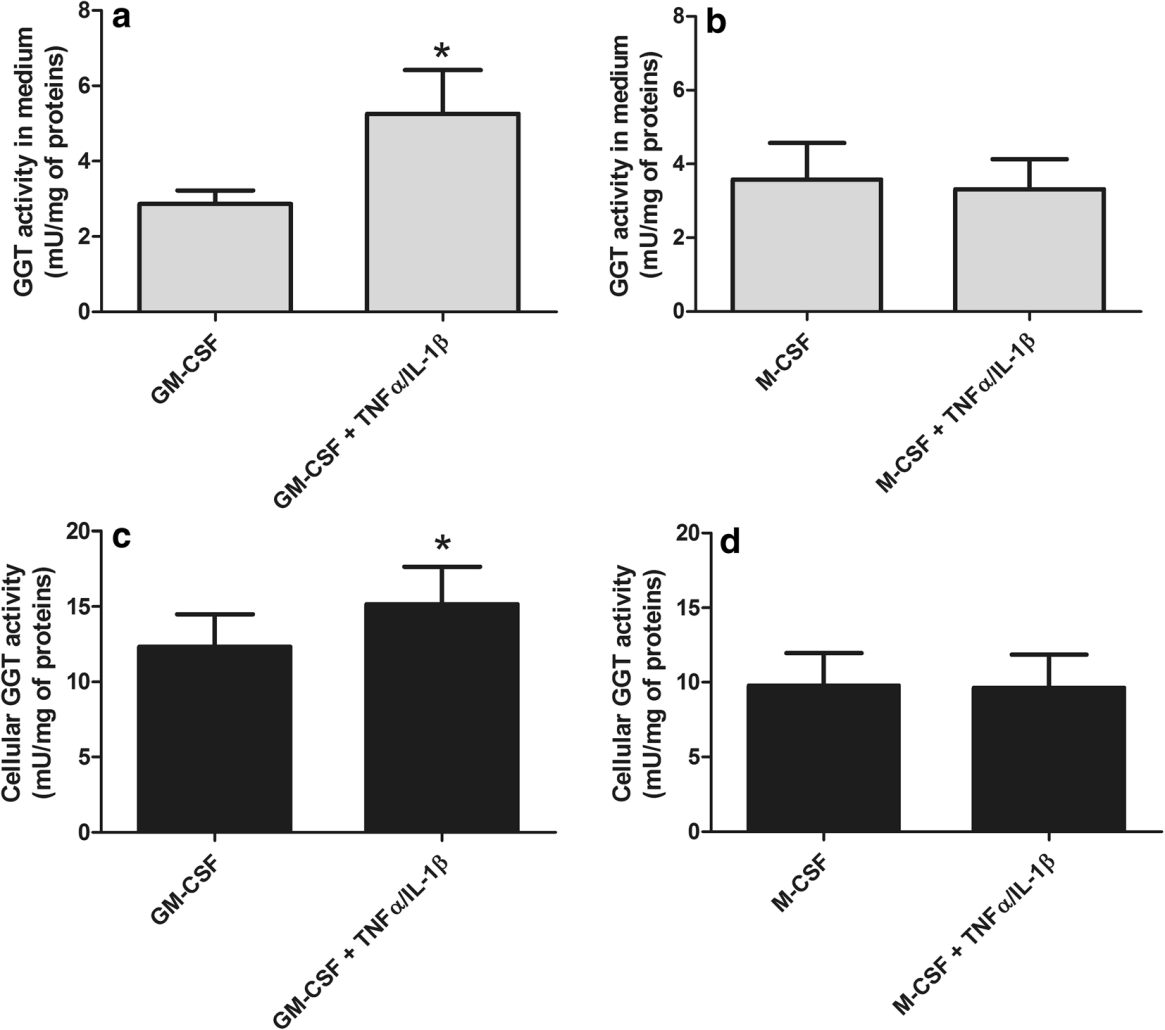
GGT release by activated macrophages. Monocytes isolated from fresh buffy coats were incubated for 6 days in complete medium in the presence of GM-CSF (50 ng/ml) or M-CSF (50 ng/ml) in order to induce the differentiation into M1-like or M2-like macrophages, then they were incubated in the presence of TNFα/IL-1β (both 10 ng/ml) for additional 24 h. GGT activity was measured in the 10,000×*g* centrifuged supernatants (**a**, **b**) and in cellular homogenates (**c**, **d**). Results are mean ± SD of eight separate determinations. Data are expressed as mU/mg of cellular proteins and were analyzed by Student’s t test for paired data; *p < 0.05 as compared with the corresponding untreated control

The exposure to pro-inflammatory cytokines TNFα/IL-1β induced a significant increase (p < 0.05) of both released (Fig. 4a) and cell-associated GGT (Fig. 4c) only in M1-like macrophages, whereas no appreciable effect was detectable in M2-like cells (Fig. 4b, d).

### Regulation of GGT expression in M1-like and M2-like macrophages

**Fig. 5:**
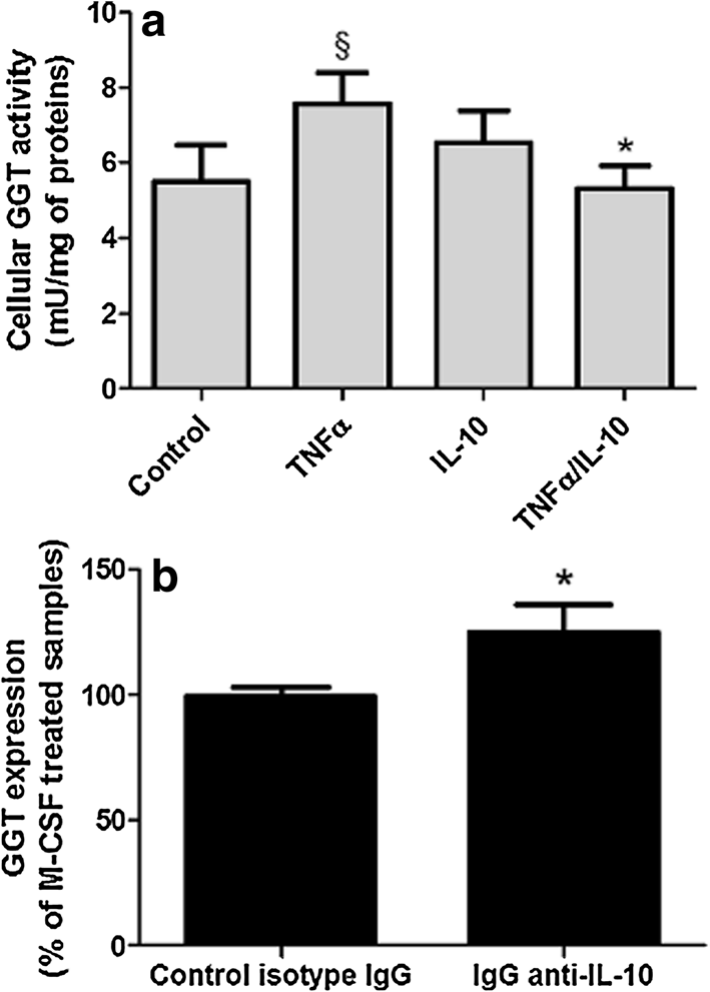
Effects of TNFα and IL-10 on GGT expression in monocytes. **a** Freshly isolated monocytes were incubated with TNFα (10 ng/ml) and/or IL-10 (20 ng/ml) in complete medium for 48 h. Results are mean ± SD of eight separate determinations. Data are expressed as mU/mg of cellular proteins and were analyzed by one-way ANOVA with Newman–Keuls test for multiple comparisons; ^§^p < 0.001 as compared to untreated control; *p < 0.001 as compared with TNFα-treated samples. **b** Isolated monocytes were let to adhere for 24 h, then they were incubated with M-CSF (50 ng/ml) in the presence of an anti-IL10 antibody (1 μg/ml) or the corresponding isotype control for 6 days. Results are expressed as the *ratio* of GGT expressed by antibody-treated cells against M-CSF treatment alone (mean ± SD of seven separate determinations). Data were analyzed by Student’s t test for paired data; *p < 0.05

In order to characterize the possible mechanisms underlying the lower expression of GGT in M2-like macrophages, we evaluated the involvement of IL-10. As expected, 48 h incubation of monocytes with TNFα resulted in increased levels of cellular GGT activity (Fig. 5a) and protein (data not shown), but this effect was completely abolished when also IL-10 was added to incubation media (Fig. 5a). In a separate set of experiments, M2-like macrophages were incubated during their differentiation with an anti-IL-10 antibody. As shown in Fig. 5b the addition of the blocking antibody was associated with induction of significantly higher levels of both GGT activity (p < 0.05) and protein (data not shown).

### Release of b-GGT fraction in monocytes/macrophages supernatants

**Fig. 6:**
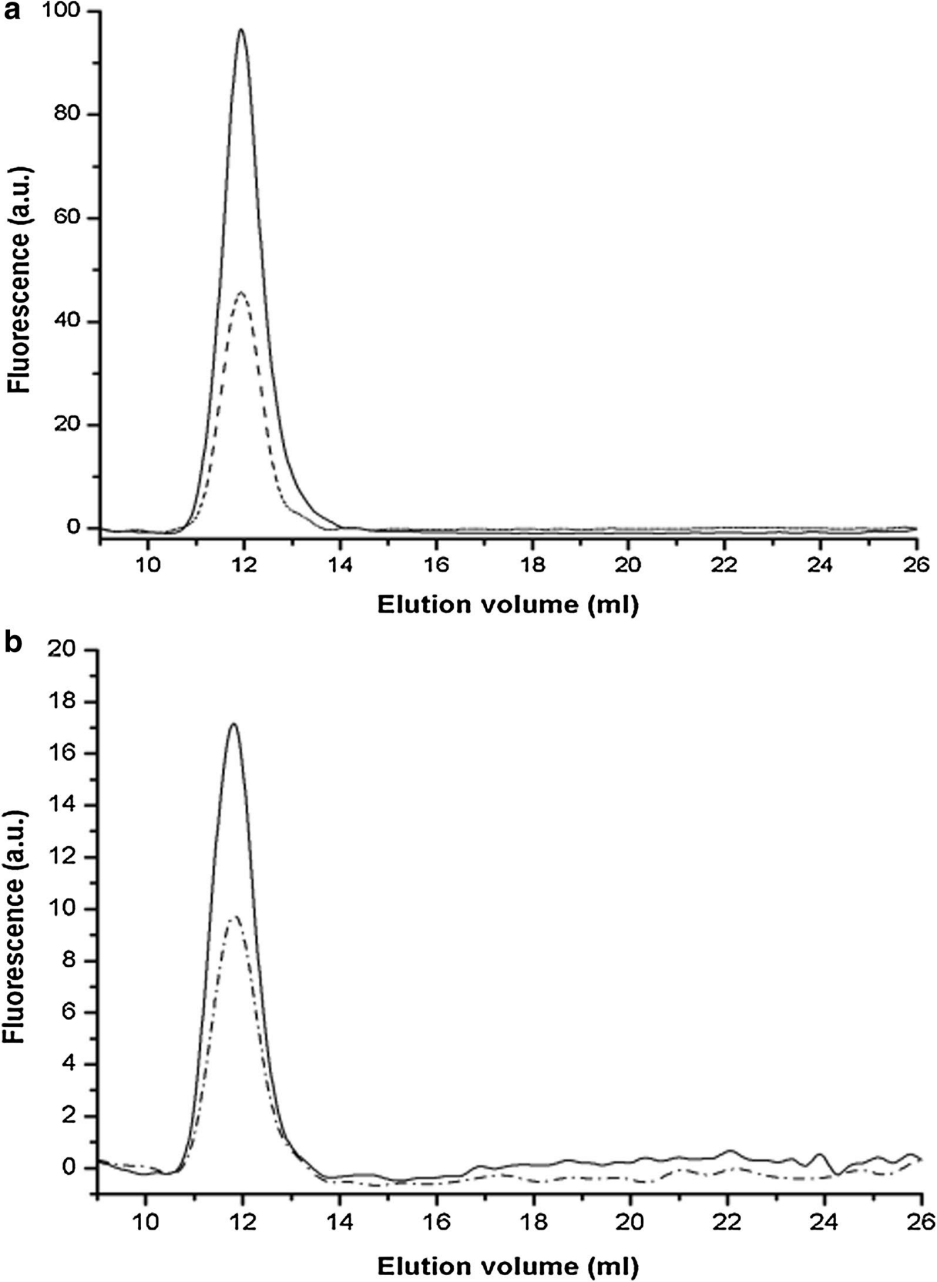
Gel filtration chromatography of b-GGT released by TNFα/IL-1-activated monocytes (**a**) and M1-like macrophages (**b**). Monocytes and GM-CSF-differentiated M1-like macrophages were incubated with TNFα/IL-1β (both 10 ng/ml, 24 h), then supernatans were pooled and ultracentrifuged (100,000×*g*, 120 min, 4 °C). The resuspended final pellets were analyzed by gel filtration chromatography. Representative elution profiles of controls (*dashed lines*) and TNFα/IL-1β- activated cells (*continuous line*) are shown

Incubation media from activated monocytes and M1-like macrophages were ultracentrifuged at 100,000×*g* and incubation media were analyzed by gel-filtration chromatography. As shown in Fig. 6 a peak corresponding to the b-GGT fraction was detected in the incubation media of both monocytes (Fig. 6a) and M1-like macrophages (Fig. 6b). In both cases, the exposure to the pro-inflammatory cytokines TNFα/IL-1β increased the release of b-GGT.

### GGT expression in atherosclerotic plaque

**Fig. 7:**
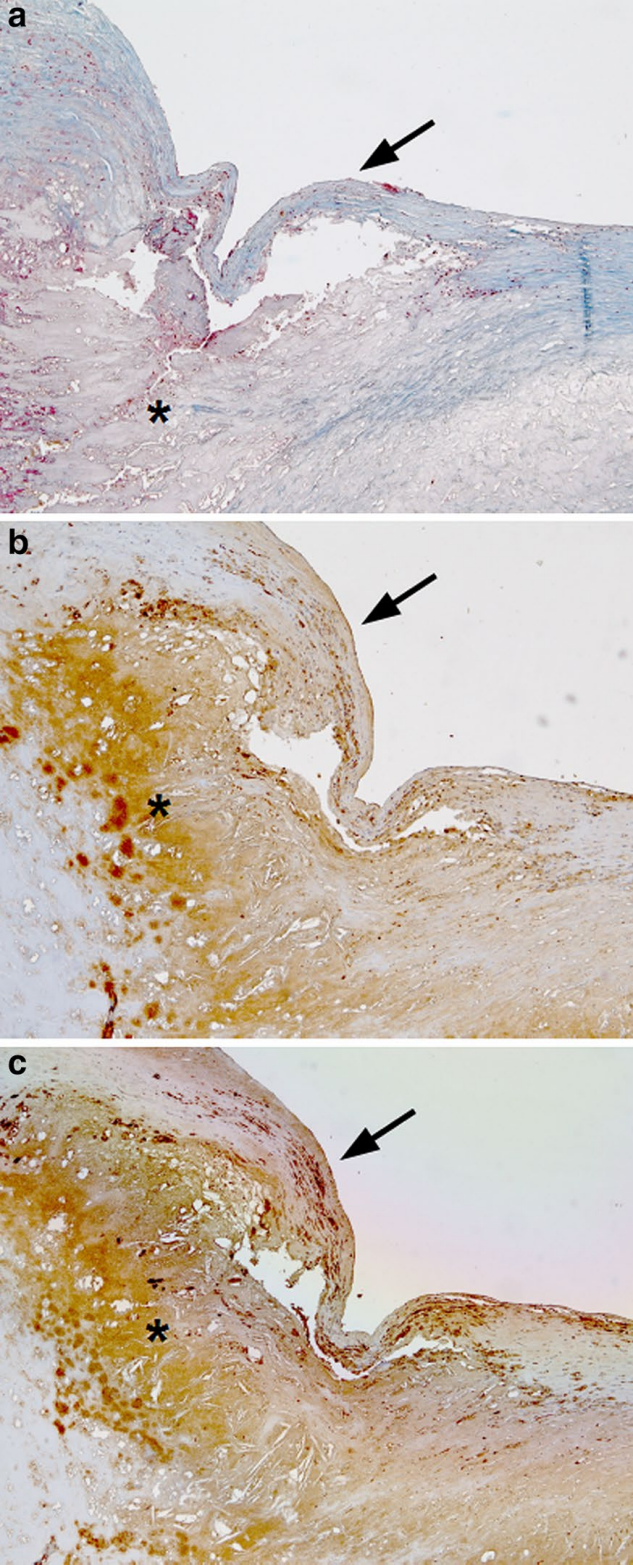
GGT expression in atherosclerotic plaques. A representative thin cap fibroatheroma without ulceration is shown. Masson’s trichrome staining (**a**; *blue* collagen fibers; *red* cytoplasm; *black* nuclei); immunohistochemistry for GGT protein (**b**) and macrophage-associated antigen CD68 (**c**). GGT and CD68 were localized by immunoperoxidase reaction with 3-diaminobenzidine as substrate, yielding a* reddish-brown* color. Immunoreactivities are mainly localized in the necrotic core (*asterisk*) extending just beneath the endothelial lining of the plaque (*arrow*). Sections were couterstained with haematoxylin. Original magnification: 10x

In agreement with previous evidence [26, 27], the immunohistochemical staining of plaques sections revealed the presence of variable amounts of immunoreactive GGT, mainly localized to areas corresponding to necrotic core and macrophage-rich sites (Fig. 7).

**Fig. 8:**
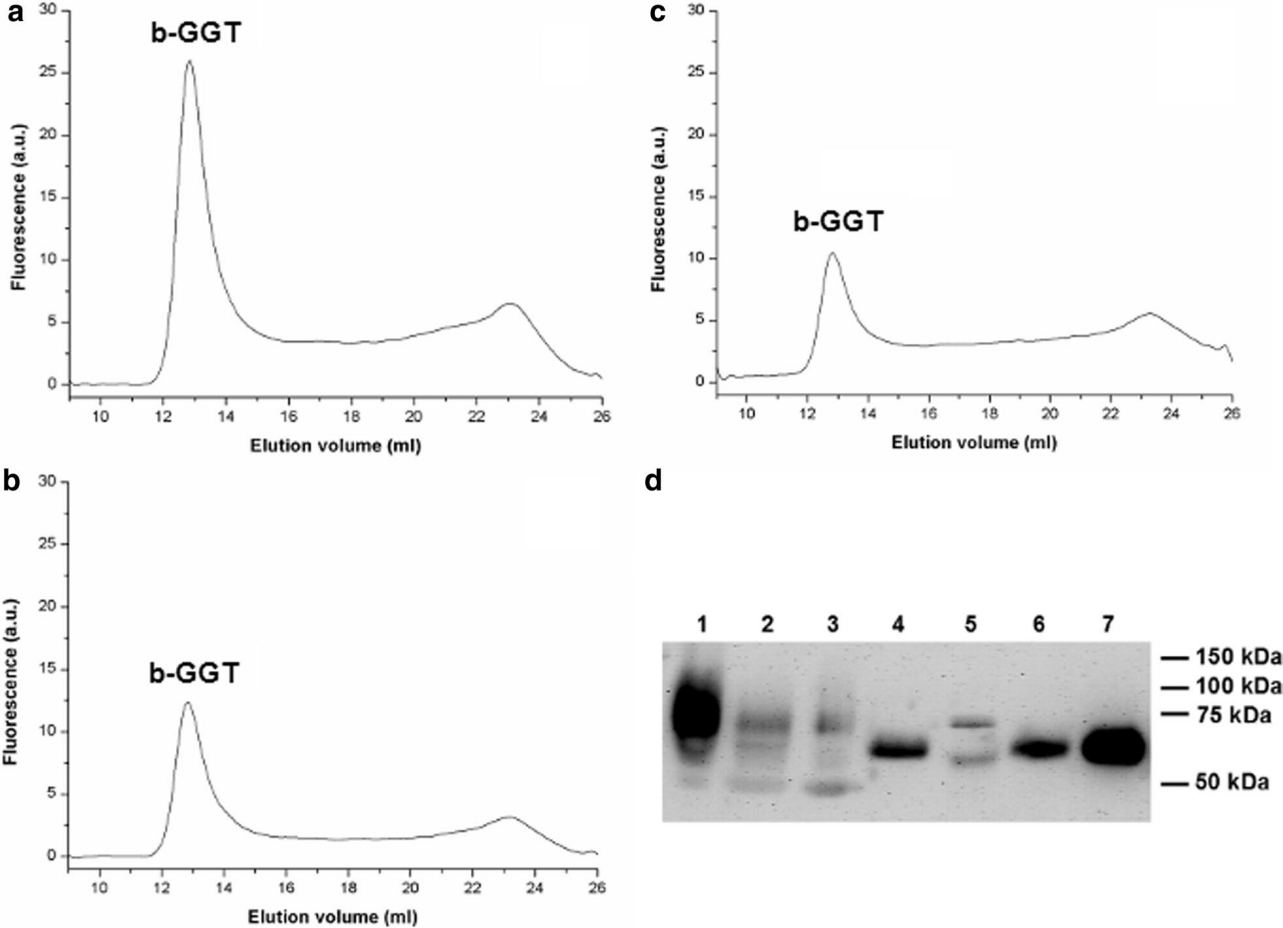
Elution profile of GGT activity from whole homogenate of three selected plaque samples (**a**, **b**, **c**). Elution profile was obtained by molecular exclusion chromatography, and GGT activity was detected by an online post-column reaction in the presence of a fluorescent substrate. A major peak of activity eluting at 12.9 ml and corresponding to the b-GGT fraction is detectable. **d** Western blot analysis of GGT heavy chain in samples of isolated plaque b-GGT and other biological samples. *Lanes 1–3* b-GGT from plaques #1–3, respectively; *lane 4* monocytes; *lane 5* HMEC-1 endothelial cells; *lane 6* lymphocytes; *lane 7* platelets

The fractional GGT analysis of plaque homogenates (Table 1) confirmed the presence of a major peak corresponding to the b-GGT fraction (Fig. 8a–c). In order to further characterize the properties of such fraction, plaque b-GGT was isolated by ultracentrifugation from all the selected samples, and the GGT protein was compared by SDS-PAGE with that expressed by monocytes, lymphocytes, platelets and endothelial cell line HMEC-1 (Fig. 8d). Using a specific antibody against the heavy subunit of GGT, a single GGT band with the same apparent molecular weight was observed in monocytes, lymphocytes and platelets, while two bands were present in HMEC-1 cells, a heavier one and one slightly lighter than those observed in the other cell types. Plaques homogenates showed three bands: one comparable with the heavier band of HMEC-1, one with the same apparent MW of monocytes, lymphocytes and platelets, and one lighter than all the other observed bands. Interestingly, the second band was clearly detectable only in samples with a higher macrophage score (#1 and #2).

## Discussion

GGT activity is a well-established independent risk factor for coronary and cerebrovascular events both in unselected populations [11] and in patients with diagnosed coronary artery disease [12]. GGT activity has been detected inside human atherosclerotic plaques associated both to CD68+ macrophage-derived foam cells [25, 26] and to a microvesicles-like fraction called b-GGT [16, 27]. The b-GGT fraction is the heaviest of the four GGT fractions identified in plasma [38] and it is the sole identified within plaque homogenates [27]. In a recent study b-GGT has been demonstrated to consist of membrane microvesicles [15], sharing the physical properties of exosomes. Interestingly, intra-plaque b-GGT activity correlates with the histological markers of plaque vulnerability—such as high macrophagic infiltration—as well as with levels of plasma b-GGT [17–27]. These pieces of evidence have prompted the need of identifying the origin of GGT accumulating inside atherosclerotic plaques, in order to better understand its role in plaque instability.

It was proposed that part of b-GGT present in atherosclerotic plaques might derive from the bloodstream through damaged endothelium [16, 27]. On the other hand an endogenous release of b-GGT by cellular elements of plaque—such as macrophages—is also conceivable. Indeed our results demonstrate that both monocytes (Figs. 1, 6) and pro-inflammatory M1-like macrophages (Figs. 4, 6) are able to release b-GGT upon activation. These results are in agreement with what already observed for neutrophils—whose contribute to inflammatory exudate b-GGT in cystic fibrosis airways has been assessed [37]—and adds evidence in support of the connection between GGT and inflammation. GGT is expressed by inflammatory cells [24] and its expression is induced by inflammatory cytokines (e.g. TNFα, IFNs) and oxidative stress. Two major inflammatory mediators, cysteinyl-leukotriene LTC4 and S-nitrosoglutathione, are GGT substrates, and it was even proposed that soluble GGT may effect a cytokine-like function [40].

The potential contribution of macrophages to plaque b-GGT accumulation was further investigated by SDS-PAGE analysis of samples belonging to a previously characterized series of carotid plaques [27]. A single peak corresponding to b-GGT was detected in all the plaques studied (Fig. 8a–c), but SDS-PAGE analysis revealed a heterogeneous composition of such fraction (Fig. 8d). In this respect it should be considered that GGT protein is transcribed from a single gene (*ggt1*), nevertheless several distinct glycosylation forms have been described, resulting in different molecular weights [41]. It was also demonstrated that inflammatory cells present in the vessel wall might induce modifications in protein sialylation status [42]. The presence of different bands corresponding to distinct MWs (Fig. 8d) may thus reflect both a heterogeneous origin for intra-plaque b-GGT as well as an intra-plaque modification of GGT protein (e.g. proteolysis, sialylation). SDS-PAGE analysis nevertheless confirmed that inflammatory as well as endothelial cells might contribute to b-GGT accumulation within the atherosclerotic plaque. SDS-PAGE does not allow discriminating among different “inflammatory” sources for b-GGT, and it is conceivable that macrophages, lymphocytes and platelets all can contribute to intra-plaque b-GGT. In particular, the demonstrated correlation between intra-plaque b-GGT activity and the levels of macrophages infiltration [27] and the fact that both monocytes (Figs. 1, 6) and M1-like macrophages (Figs. 4, 6) are able to release b-GGT upon activation, suggest that macrophages play a major role in b-GGT accumulation inside the atherosclerotic plaque.

As previously observed for neutrophilic infiltrates in cystic fibrosis [37], b-GGT accumulation in the atherosclerotic plaque should not be interpreted as the mere epiphenomenon of an exogenous contribute (i.e., from the bloodstream), or as the effect of inflammation-related oxidative stress. Rather, the expression and the release of b-GGT by macrophages may be considered as one of the effects associated with the immune response, possibly involved in the modulation of selected mediators (*e.g.* GSNO, leukotrienes). Moreover, in selected conditions GGT activity has been shown to promote the production of reactive oxygen species and other prooxidants, resulting in redox reactions potentially involved in the progression of atherosclerosis [19–21]. M1 macrophage content of atherosclerotic plaques is positively associated with increased inflammation and clinical incidence of ischemic stroke [9]. High numbers of M1 macrophages were detected in symptomatic plaques, whereas smaller numbers, predominantly staining for M2 macrophage markers, were detected in asymptomatic plaques [5, 7–9]. The higher levels of GGT expression and b-GGT release observed from M1-like macrophages may thus play a role in plaque instability.

Altogether, our data indicate that inflammatory conditions are able to increase the expression and release of GGT enzyme, which in turn could play a role in modulating inflammation in the plaque environment. The higher cellular GGT expression in GM-CSF-induced M1-like macrophages, as compared to M-CSF-induced M2-like ones (Figs. 1a, b), appears to further support such hypothesis. Our data are in agreement with earlier evidence obtained with the human leukemic cell line KG-1, where incubation with GM-CSF resulted in a significant increase in GGT enzyme activity, while M-CSF did not produce any appreciable effect [43]. As far as treatment of monocytes with GM-CSF and M-CSF it has been reported that both GM-CSF and M-CSF receptors activate Ras-dependent signal transduction pathways and are both capable of activating AP-1 [44]. GM-CSF [45] and to some extent M-CSF [46] are also known to modulate NF-κB activation, but with some important differences in terms of timing, duration and factors involved [47]. In agreement with previous evidence [33, 47], our M1-like macrophages produced higher levels of pro-inflammatory TNFα and IL-6 and lower levels of anti-inflammatory IL-10, whereas the opposite was observed with M2-like macrophages (Fig. 2). Indeed, it has been proposed that Ras signalling is involved in GGT expression [48], and synthesis of GGT mRNA is induced by cytokines—including TNFα—through a NF-κB-dependent pathway [49, 50]. The differential basal expression of cytokines in M1-like and M2-like macrophages could thus account for the differential GGT expression observed. Indeed TNFα was able to significantly increase GGT expression in monocytes, but this effect was inhibited in the presence of IL-10 (Fig. 5a), i.e. a cytokine known to inhibit NF-κB pathway [51, 52]. Accordingly, when monocytes were differentiated into M2-like cells, the addition of an anti-IL-10 antibody was associated to higher levels of GGT expression (Fig. 5b). The lack of GGT induction in M2-like cells following TNFα/IL-1β might be therefore explained by such inhibitory effect of endogenous IL-10 (Fig. 4c, d), whose expression is markedly higher in M2- as compared to M1-like cells [47]. These observations support the hypothesis that the balance of pro-inflammatory/anti-inflammatory cytokines such as TNFα and IL-10 may differentially modulate GGT expression.

Besides GM-CSF or M-CSF treatments, macrophage M1 or M2 phenotypes have also been obtained by other in vitro procedures. M-CSF was shown to induce M1 differentiation in the presence of IFN-γ and/or bacterial products such as LPS [53], whereas three distinct M2 subtypes were induced by treatments with IL-4 or IL-13 (“M2a” phenotype), or immune complexes in combination with IL-1β or LPS (“M2b”), or IL-10, TGFβ and glucocorticoids (“M2c”) [54]. Interestingly, the *ggt5* gene (formerly designated as GGTLA1/GGT-rel or GGL) was also recently shown to be highly expressed in GM-CSF differentiated macrophages [33]. GGT5 is a member of GGT family supposed to be mainly responsible for the conversion of LTC4 to LTD4 [55].

## Conclusions

The results of the present study indicate that macrophages characterized by a pro-inflammatory phenotype can release b-GGT and contribute thus to its accumulation inside atherosclerotic plaques. Our observations may explain the existing association between b-GGT activity in thin-cap fibroatheromas and greater macrophage infiltration [27]. Further studies are however needed to fully identify the different sources of intra-plaque b-GGT and their actual role in the pathogenesis/progression of atherosclerosis.

## Abbreviations

AP-1: activator protein 1
b-GGT: big-gamma-glutamyltransferase
FPLC: fast protein liquid chromatography
GGT: gamma-glutamyltransferase
GM-CSF: granulocyte/macrophage colony stimulating factor
HMEC-1: human dermal microvascular endothelial cells
IL: interleukin
LPS: bacterial lipopolysaccharide
M-CSF: macrophage colony stimulating factor
NF-κB: nuclear factor kappa-light-chain-enhancer of activated B cells
PBMCs: peripheral blood mononuclear cells
TNFα: tumor necrosis factor alpha
